# Tea Not a Food but by Order

**Published:** 1918-05-11

**Authors:** 


### KING'S BENCH JUDGES AT TEA.

### Tea Not a Food but By Order.

Three judges, Mr. Justice Darling, Mr. Justice Avory, and Mr. Justice Shearman, have been taking tea in the Court of King's Bench, and have pronounced upon it a decision that must carry consternation to the heart of every teetotaller. Their lordships have decided that tea is not a food, and thus have turned against the favourite beverage of the teetotaller the very argument that he adduces with such "damnable iteration" against alcohol. We ought not, so the teetotaller argues, to take wine with our dinner or beer with our lunch, for alcohol, which, as every teetotaller knows, is the only constituent of beer and wine, is not a food. The teetotaller should, if he possessed any logical faculty at all, forbid us also to eat beef, or mutton, or bread, since none of these substances is a beverage; but whatever the virtues of the teetotaller, not even his best friends will assert that a capability of appreciating logical consequences is amongst them. He is now confronted with the appalling fact that the very same argument that he has been bringing so monotonously and for so many years against alcohol is applicable to tea also; and is, in fact, much more applicable; for the highest authorities are now agreed that alcohol does act as a food. It does contain a small, but quite appreciable, proportion of food. But tea, alas! contains none at all. So the Court of King's Bench has decided, and every dietetic expert will say Amen.

> But soon a wonder came to light,
> Which showed the rogues they lied.

Far be it indeed from us to apply an epithet so opprobious to gentlemen of such distinguished rectitude; but still, there is undeniably a certain aptness in the quotation, a certain applicability, which we regret and deprecate, even while we recognise it; for hot-foot upon the decision of the Divisional Court comes a decision of the Privy Council, which reverses the Court of King's Bench, and declares that for the purposes of the Food Hoarding Order, food shall include tea.

### The Food Controller's Logic.

It behoves us to move warily in criticising authorities of such dazzling eminence as the Court of King's Bench and His Majesty's Privy Council; but, after all, logic is logic, and is no respecter of persons, however respectful such humble exponents of it as ourselves may desire to be. The Privy Councillor who promulgates the amendment to the Food Hoarding Order is not content merely to declare that for the purposes of that Order tea shall be considered a food. Whether by way of rebuking the Court of King's Bench for its temerity in giving a decision on a point of law, and incidentally of common sense also, or whether from a laudable but futile desire to impart an air of reasonableness to its declaration, the authority that promulgates the amendment to the Food Hoarding Order gives what seems to be intended as a justification or explanation of its amendment. It says "The expression 'article of food' shall mean every article which [the authority means "that" but it says "which"] is used as food by man, and every article which [again the same mistake] ordinarily enters into or is used in the composition or preparation of human food; and shall include tea, coffee, and cocoa." Evidently the intention is to show that tea ought to have been recognised as included in food, and would be so included if the word "food" were rightly interpreted. But this does not follow. Plainly and manifestly it does not follow. Tea is not an article that is used as food by man. The Court of King's Bench decided that it is not, and every authority on dietetics will endorse its decision. Nor does tea enter into the composition or preparation of human food. It may be plausibly asserted that coffee does so, for coffee is occasionally made into a mould with cornflour or some other farinaceous substance, but tea never. Nor is tea ever used in the preparation of human food. No cook washes her vegetables in tea, or uses tea to boil her puddings in. No. Look at it how we may, strain the English language to its utmost limits, and still we cannot bring tea under any reasonable description of food. The Privy Councillor would have been much better advised to content himself with the bare direction that for the purpose of the Food Hoarding Order tea should be included in food. Here he is within his rights. Here no question of logic is involved. His powers are plenary. He can, if he chooses, declare that sawdust and millstones shall, for the purpose of the Food Hoarding Order, be included in food; but there are certain restrictions to the power even of the Food Controller. Except for the purposes of the Food Hoarding Order, he cannot make that a food which is not a food, nor, we may add, can he make that good English which is not good English; but we have long ceased to expect anything but execrable English in official publications.

